# Nutrition and Allergic Diseases

**DOI:** 10.3390/nu9070762

**Published:** 2017-07-17

**Authors:** R. J. J. van Neerven, Huub Savelkoul

**Affiliations:** 1 Wageningen University & Research, Cell Biology and Immunology, 6709 PG Wageningen, The Netherlands; huub.savelkoul@wur.nl; 2 FrieslandCampina, 3818 LE Amersfoort, The Netherlands; 3 Allergy Consortium Wageningen, 6709 PG Wageningen, The Netherlands

**Keywords:** asthma, rhinoconjunctivitis, eczema, (food) allergy, nutrition, fatty acids, breastfeeding, pre/probiotic

## Abstract

The development of IgE-mediated allergic diseases is influenced by many factors, including genetic and environmental factors such as pollution and farming, but also by nutrition. In the last decade, substantial progress has been made in our understanding of the impact that nutrition can have on allergic diseases. Many studies have addressed the effect of breastfeeding, pre-, pro- and synbiotics, vitamins and minerals, fiber, fruit and vegetables, cow’s milk, and n-3 fatty acids, on the development of allergies. In addition, nutrition can also have indirect effects on allergic sensitization. This includes the diet of pregnant and breastfeeding women, which influences intrauterine development, as well as breastmilk composition. These include the diet of pregnant and breastfeeding women that influences intrauterine development as well as breastmilk composition, effects of food processing that may enhance allergenicity of foods, and effects via modulation of the intestinal microbiota and their metabolites. This editorial review provides a brief overview of recent developments related to nutrition and the development and management of allergic diseases.

## 1. Introduction

Approximately 10% of children without an allergic parent or sibling, and 20% to 30% of those with allergies in their first-degree relatives, experience allergic diseases in infancy. Asthma, rhinoconjunctivitis and eczema are three prevalent non-communicable diseases, which are caused by allergy. The prevalence varies between and within countries. Globally, the prevalence for current asthma, rhinoconjunctivitis and eczema in the 13–14-year age group has been reported to be 14.1%, 14.6% and 7.3%, respectively. In the 6–7-year age group, the prevalence for current asthma, rhinoconjunctivitis and eczema has been reported to be 11.7%, 8.5% and 7.9%, respectively [1]. Currently, 8.4% of persons in the United States have asthma as compared with 4.3% of the population worldwide (300 million people), and both numbers are on the rise. The sharp rise in the prevalence of asthma was first noted in the western world, but other regions are now following the same trend [2,3,4]. Likewise, the prevalence of food allergy is also increasing [5,6]. Studies based on oral food challenges indicate that the prevalence of food allergy amongst preschool children is currently between 5% and 10% in some western countries (e.g., UK, Australia), and 7% in China, based upon a combination of clinical history and measurement of sIgE [7]. The prevalence of food allergy now ranges between 3% and 35% in self-reported studies, being lower (2–5%) when assessing for sensitization and symptoms to food. The prevalence of food allergy is even lower when double blind placebo controlled food challenges (DBPCFC) are performed, but only a limited number of studies have used this method to confirm the diagnosis of food allergy [8].

**Figure 1:**
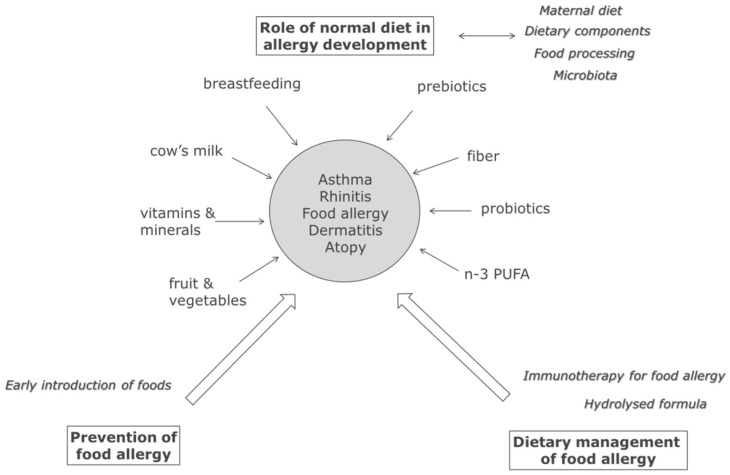
Schematic overview of the influence of nutrition on allergic disease. Nutrition can—in addition to genetic and environmental factors—play an important role in allergic diseases. Dietary components present in a normal diet may contribute to prevention of allergies (asthma, rhinitis, dermatitis, atopy and food allergies), promote the development of allergies (food processing, food allergy), and more specialized foods can be used for the management or even the treatment of food allergy.

It is clear that the development of IgE mediated allergic diseases is influenced by many factors, including genetic and environmental factors such as pollution and farming, and also by nutrition. Nutrition can affect the development of allergies during intrauterine development, after birth during breastfeeding or bottle feeding, and later after weaning when other foods are introduced. In addition, food can also be used as a tool to actively prevent (via timing of introduction) manage (hydrolyzed formula), or even treat (immunotherapy) food allergy (Figure 1).

The purpose of this special issue of Nutrients on nutrition and allergic diseases is to provide an overview of how nutrition can modify allergies. More specifically the issue addresses the influence of nutrients and foods present in a normal diet on the development of allergies—and via which mechanisms they can induce these changes. Several reviews and original papers address these questions. In this editorial review we will briefly touch upon recent developments in the field to introduce and position some of the papers in this special issue.

## 2. Maternal Diet, Breastfeeding, and Infant Nutrition & Allergy

The first 1000 days of life are crucial in the growth and development of infants. Especially during the first year they have a diet of limited variability, mainly consisting of breastmilk and/or infant formula, followed by the introduction of normal milk and solid foods. As a consequence, the composition of these foods has a more prominent effect on immune development than later in life, when the diet is more varied and the immune system has already matured.

The first contact of infants with nutrition is after conception, when the nutritional status of the mother can already have an impact on intrauterine development of the fetus. In addition, maternal diet can also influence breast milk composition. Several papers in this issue focus on the associations between maternal diets with allergy development later in life. McStay et al. reviewed studies on folic acid supplementation in pregnant women [9], and noted that folic acid intake may be linked to childhood allergic disease. On the other hand, other maternal diet components, such as poly unsaturated fatty acids, probiotics, and prebiotics, may have a protective effect on allergy development [10,11,12,13]. The review by Miles in this issue provides an overview of the current knowledge on the intake of fish-derived polyunsaturated fatty acids during pregnancy and atopic eczema in the first year of life [14].

Several, but not all, studies on the association between breastfeeding and allergy have shown effects on allergic outcomes [15,16,17]. One of the factors that may explain the conflicting findings described above may be the result of differences in breastmilk composition [18]. For example, higher levels of TGF-β in breast milk have been reported to be associated with lower allergy prevalence [19,20,21,22], although also here there are conflicting reports that did not find this association [20,23]. In this issue, a review and an original study discuss the relationship between breastfeeding, human breast milk composition, and the development of allergic diseases [24,25].

When avoidance of food allergens is not possible, as is the case in infants with cow’s milk allergy, hydrolyzed formula foods are used in the management of cow’s milk allergy to prevent allergic reactions. For infants that are diagnosed with cow’s milk allergy formula consisting of extensively hydrolyzed milk, soy or rice protein, as well as amino acid formulas are used as reviewed in [26]. For infants at risk of developing cow’s milk allergy, other milk formulas are available. These consist of milk proteins that are only partially hydrolyzed. As most of the IgE binding epitopes have been removed by hydrolysis, these hydrolysates may reduce the risk of developing cow’s milk allergies, although there is no scientific consensus on its efficacy yet [27,28]. This is also discussed in the review by VandenPlas et al. in this issue [29].

## 3. Modulation of Microbiota and Allergy in Early Life

The notion that composition and metabolic activity of the intestinal microbiota affects the development of allergies has become clearer over the last years [30,31,32,33].

The intestinal microbiota can be modulated by non-digestible oligosaccharides (human milk oligosaccharides in breast milk or prebiotic oligosaccharides), a fiber-rich diet, and by probiotics. The effects of pre-, pro- and synbiotics on allergies—most notably in eczema—has been the subject of many studies, reviewed in [34,35,36]. This is also the subject of the review by Hulshof in this issue, on the management of atopic dermatitis in children [37]. Likewise, Aitoro et al. discuss the potential of targeting the gut microbiota in food allergy [38].

Exactly how the microbiota composition influences allergy development is not clear at this point, but data from animal models strongly suggest a protective role for short chain fatty acids produced upon fermentation of fiber and oligosaccharides (propionate, butyrate, acetate) [11,39,40]. The molecular mechanisms behind the effect of dietary fiber on allergy via microbiota composition and metabolic activity are discussed in the review by Wypych and Marsland in this issue [41].

## 4. Normal Dietary Components and Allergy

After weaning and introduction of milk and solid foods into the diet, additional factors may prevent or contribute to the development of allergies. Essentially, all dietary antigens are proteins, and therefore highly digestible diets are recommended for food allergic individuals to reduce the number of intact antigens reaching the Peyer’s patches. Solid foods associated with lowered allergy prevalence include fruit and vegetables, vitamins, polyunsaturated fatty acids, and (raw) cow’s milk, but the processing of foods can possibly also affect allergy development [10,42,43,44,45,46,47,48,49,50]. These food components, as well as the effects of food processing, are also addressed in the papers by Hosseini et al. (fruit & vegetables), Brick et al. (milk and processing), and Teodorowicz et al. (food processing) in this issue [51,52,53].

## 5. IgE Antibody Characteristics and the Allergic Phenotype

Finally, processing of foods may influence the allergenicity of these foods [47]. This is discussed in the paper by Teodorowicz et al. in this issue [53]. Heat processing induces Maillard reactions, “gluing” carbohydrates to food proteins, which as a result become more immunogenic and probably also allergenic, thus promoting the development of IgE responses to food allergens.

At present, no identified antibody characteristics and no identified structural features of IgE binding epitopes seem to be associated with the phenotype of the food allergic disease. Recent studies have suggested that IgE directed towards linear epitopes may react with foods in processed forms (heated and digested), while IgE binding to conformational epitopes may be impaired by such processing because of changes in allergen tertiary structure. In addition, linear epitopes have been suggested to potentially be biomarkers for a persistent form of food allergy [54].

## 6. Food Allergy: Early Introduction and Immunotherapy

Even though nutritional guidelines for food allergy and treatment of food allergies are not the scope in this special issue, several developments deserve attention and will be mentioned briefly below.

Food allergy is an IgE-mediated reaction to a food, usually during the 2 h following its intake. It represents a health problem that can lead to life-threatening reactions and can even impair quality of life. Any food can potentially trigger an allergic response; in fact, more than 170 foods have been identified as being potentially allergenic, but the vast majority of the clinically diagnosed food allergies are caused by only a few of these foods. Despite relevant advances in the knowledge of food allergy during the last decades, gaps in this area are evident, especially in relation to introduction of allergenic foods and in relation to application of food immunotherapy.

As the prevalence of food allergies in many countries continues to rise, the question remains as to when to introduce specific allergenic solid foods in infants. The current consensus recommendation by allergologists is to introduce solid foods after 4 months of age to prevent food allergy. This is documented by observational studies that later introduction of solid foods is linked to an increased risk of obesity, gastrointestinal disorders and development of allergy. However, current dietary guidelines still recommend introduction of solid foods at around 6 months of age. The intent of these guidelines is to prevent replacing breastfeeding with lower energy and nutrient dense foods (certainly in malnourished communities) beyond 6 months of age, thereby inducing consequential malnutrition [55].

However, recent studies indicate that early introduction of food allergens into the diet of young children, as well as the early introduction of diverse foods may actually prevent food allergy [56,57,58], suggesting that immune tolerance can be readily induced to food allergens in early life. The results also suggest that there is no reason to delay the introduction of the allergenic foods into the infant’s diet after solid foods have started. Nevertheless, some infants are sensitized to food allergens before any known ingestion of solid foods and future research needs to focus on strategies to prevent early-life food allergen sensitization prior to complementary feeding [59].

The results from the Learning Early about Peanut Allergy (LEAP) study [56] have led to consensus statements from international pediatric, allergy and dermatology societies encouraging and recommending the early introduction of peanut butter, cooked egg, dairy and wheat products to infants at (even high) risk of developing food allergy. However, besides effectiveness, safety should also be considered when introducing potential allergens into the diet [60,61].

Finally, for people who have already developed food allergies, much has been done on the development of new immunotherapies for food allergy. Safe, specific immunotherapy is not currently available for IgE-mediated food allergy due to the high risk of anaphylaxis. Oral immunotherapy, epicutaneous immunotherapy, or sublingual immunotherapy for food allergy are increasingly being studied, and some innovative approaches have been suggested, such as modification of relevant food allergens (to make them less allergenic while maintaining their immunogenicity), or combining other non-specific treatments (e.g., probiotics) to increase efficacy and/or safety [62,63].

## 7. Conclusions

Our understanding of the influence of nutrition on allergic diseases is increasing steadily. The papers in this special issue provide an overview of current knowledge in the field and identify several of the directions in which developments are taking place.
